# C-ziptf: stable tensor factorization for zero-inflated multi-dimensional genomics data

**DOI:** 10.1186/s12859-024-05886-4

**Published:** 2024-10-05

**Authors:** Daniel Chafamo, Vignesh Shanmugam, Neriman Tokcan

**Affiliations:** 1 Perelman School of Medicine, University of Pennsylvania, Philadelphia, PA 19104 USA; 2 Broad Institute of MIT and Harvard, Cambridge, MA 02142 USA; 3 Department of Mathematics, University of Massachusetts Boston, Boston, MA 02125 USA; 4 Department of Pathology, Brigham and Women’s Hospital, Boston, MA 02215 USA

**Keywords:** Tensor decomposition, Multimodal genomics data, Bayesian inference, Zero-inflated model, Factor analysis, Multi-sample multi-condition single-cell data

## Abstract

In the past two decades, genomics has advanced significantly, with single-cell RNA-sequencing (scRNA-seq) marking a pivotal milestone. ScRNA-seq provides unparalleled insights into cellular diversity and has spurred diverse studies across multiple conditions and samples, resulting in an influx of complex multidimensional genomics data. This highlights the need for robust methodologies capable of handling the complexity and multidimensionality of such genomics data. Furthermore, single-cell data grapples with sparsity due to issues like low capture efficiency and dropout effects. Tensor factorizations (TF) have emerged as powerful tools to unravel the complex patterns from multi-dimensional genomics data. Classic TF methods, based on maximum likelihood estimation, struggle with zero-inflated count data, while the inherent stochasticity in TFs further complicates result interpretation and reproducibility. Our paper introduces Zero Inflated Poisson Tensor Factorization (ZIPTF), a novel method for high-dimensional zero-inflated count data factorization. We also present Consensus-ZIPTF (C-ZIPTF), merging ZIPTF with a consensus-based approach to address stochasticity. We evaluate our proposed methods on synthetic zero-inflated count data, simulated scRNA-seq data, and real multi-sample multi-condition scRNA-seq datasets. ZIPTF consistently outperforms baseline matrix and tensor factorization methods, displaying enhanced reconstruction accuracy for zero-inflated data. When dealing with high probabilities of excess zeros, ZIPTF achieves up to $2.4\times$ better accuracy. Moreover, C-ZIPTF notably enhances the factorization’s consistency. When tested on synthetic and real scRNA-seq data, ZIPTF and C-ZIPTF consistently uncover known and biologically meaningful gene expression programs. Access our data and code at: https://github.com/klarman-cell-observatory/scBTF and https://github.com/klarman-cell-observatory/scbtf_experiments.

## Introduction

Advancements in genomics have given rise to diverse methods for studying gene expression patterns, with single-cell RNA-sequencing (scRNA-seq) representing a pivotal milestone [1]. The advent of single-cell RNA sequencing has enabled the exploration of gene expression at the single-cell level, revealing cellular heterogeneity and capturing rare cell types [2]. Simultaneously, multi-sample and multi-condition single-cell studies have emerged, leveraging scRNA-seq to analyze multiple samples under differing conditions [3]. Applications of these datasets span disease comparisons, drug response studies, temporal cellular analyses, and more. These studies provide insights into the variability and robustness of molecular signatures and identify condition-specific patterns that transcend individual samples.

The upshot of these developments has been an unprecedented surge in multidimensional genomic data and an increasing demand for robust methodologies capable of navigating the complexity inherent in such data [4]. More recently, analysis methods that treat individual cells as statistically independent replicates have faced scrutiny as they overlook the correlation between cells originating from the same individual [5, 6]. An increasingly popular solution is the use of pseudobulk aggregates—gene counts averaged across individual and cell type—as the basis of downstream analysis. Tensors, which are multi-way arrays that extend matrices to higher dimensions, provide a natural way to represent this data. Examining gene variations across multi-condition samples and various cell types leads to a 3-way tensor (samples $\times$ cell types $\times$ genes). Tensor factorization methods have emerged as powerful tools offering unique capabilities to unravel complex patterns from such multi-dimensional data in an unsupervised manner [7–9]. While it is possible to use traditional matrix methods on pseudobulk data, this would require *matricizing* tensors, limiting the potential to exploit the intrinsic multi-way structure of the data [10]. Tensor factorization extends matrix factorization to higher dimensions while preserving intrinsic structure, enabling the discovery of complex interactions.

Alongside high-dimensionality, another significant challenge in single-cell genomics data is its sparsity. Single-cell data is sparse due to low capture efficiency during sequencing and “dropout events” in which lowly expressed genes remain undetected [4]. This sparsity, coupled with high-dimensional gene expression patterns across diverse cells, complicates analysis and interpretation of single-cell data. Classic tensor factorization methods using maximum likelihood estimation (MLE) can be unreliable when applied to sparse count data [11]. Bayesian Poisson Tensor Factorization (BPTF)—a higher-order extension of Poisson matrix factorization—overcomes the limitations of the MLE approach when dealing with high-dimensional count data. BPTF provides advantages such as the ability to incorporate prior knowledge, perform model selection, and quantify uncertainty in parameter estimates [12–14]. Highly-dispersed count data with excess number of zeros is common in various fields beyond genomics, including healthcare (e.g., hospital readmissions), social sciences (e.g., user behaviors), and insurance claims [15, 16]. The Zero-Inflated Poisson (ZIP) distribution is a better model for such data compared to the Poisson distribution [15, 17, 18], and has been successfully used in recommendation systems and other applications [16].

In addition to modeling the distribution of data and noise appropriately, another issue to be addressed is the inherent randomness of tensor factorization algorithms. This leads to varying results for multiple runs and negatively impacts the interpretability and reproducibility [10, 19]. In this paper, we propose a novel approach for stable tensor factorization which is robust for high-dimensional sparse count data with excess zeros (Section Materials and methods). We claim three **main contributions**:

- We propose a novel factorization approach for high-dimensional sparse count data with excess zeros, namely *Zero Inflated Poisson Tensor Factorization (ZIPTF)*, which utilizes a Bayesian ZIP model (Section Materials and methods).
- To address the discussed randomness issue, we develop a meta-analysis method that generalizes consensus matrix factorization [20] and incorporates novel techniques to improve the stability and interpretability of the factorization results (Section  Generic consensus-based tensor factorization, Fig. 2). We specifically focus on its integration with ZIPTF, namely *Consensus-ZIPTF (C-ZIPTF)*. Nonetheless, our method is generalizable to other factorization approaches.
- We provide an extensive evaluation on four different datasets: (1) synthetic zero-inflated count tensors with increasing probability $\Phi$ of excess zeros (Section  Synthetic tensor experiment); (2) synthetic multi-sample scRNA-seq data (Section  Synthetic single-cell RNA-Seq data analysis); (3) real multi-sample, multi-condition scRNA-seq dataset of immune cells stimulated with interferon beta (Section  Identification of cell type identity and perturbation-specific programs using C-ZIPTF in single-cell RNA-seq data); (4) real scRNA-seq data collected from patients with systemic lupus erythematosus (including those with managed and active flare) and healthy groups (Section  C-ZIPTF enables unsupervised discovery of disease subgroups and multicellular gene expression programs in the peripheral blood of patients with systemic lupus erythematosus).

We compare ZIPTF and C-ZIPTF against baseline matrix and tensor factorization approaches, as well as established scRNA-seq data analysis methods. Our results indicate that ZIPTF outperforms the baselines in terms of reconstruction accuracy for zero-inflated data. Specifically, for $\Phi =0.8$, ZIPTF achieves an average explained variance of 0.92, compared to a maximum of 0.38 achieved by the baseline models. Additionally, C-ZIPTF significantly improves the consistency and accuracy of the factorization results. Both ZIPTF and C-ZIPTF successfully capture biologically meaningful gene expression programs (GEPs) and result in factors with higher Pearson correlations to known GEPs. Finally, C-ZIPTF adeptly captures condition-specific GEPs, unveiling nuanced expression patterns that highlight intra-group heterogeneity, a facet often overlooked by supervised methods like Differential Gene Expression (DGE) Analysis.

## Tensor preliminaries

This section presents the foundational concepts and notations for tensors, with most of the notation borrowed from [10]. We denote the $(i_1, i_2, \ldots , i_N)$-th entry of an *N*- way tensor ${\mathcal {X}} \in {\mathbb {R}}^{I_1 \times I_2 \times \ldots \times I_N}$ as ${\mathcal {X}}_{i_1i_2\ldots i_N}$. The Frobenius norm of a tensor is similar to the matrix Frobenius norm:

1 $$\begin{aligned} \Vert {\mathcal {X}}\Vert_{F}= \sqrt{\sum _ {i_1=1}^{I_1}\sum _{i_2=1}^{I_2}\ldots \sum _{i_N=1}^{I_N}{\mathcal {X}}_{i_1i_2\ldots i_N}^2}. \end{aligned}$$

An *N*-way tensor ${\mathcal {Y}}$ is called a rank-1 tensor if it can be written as outer product of *N* vectors, i.e., ${\mathcal {Y}}=u^{(1)}\otimes u^{(2)} \otimes \ldots \otimes u^{(N)}$ with ${\mathcal {Y}}_{i_1i_2\ldots i_N}= u_{i_1}^{(1)}u_{i_2}^{(2)}\ldots u_{i_N}^{(N)}$. A rank $R\ge 1$ approximation to the tensor ${\mathcal {X}} \in {\mathbb {R}}^{I_1 \times I_2 \times\ldots\times I_N}$ can be given as:

2 $$\begin{aligned} {\mathcal {X}} = \mathcal {\tilde{X}} + {\mathcal {E}}~\text {where}~ \mathcal {\tilde{X}}= \sum _{r=1}^{R} a^{(1)}_{r}\otimes a^{(2)}_{r} \otimes \dots \otimes a^{(N)}_{r}, \end{aligned}$$

**Fig. 1:**
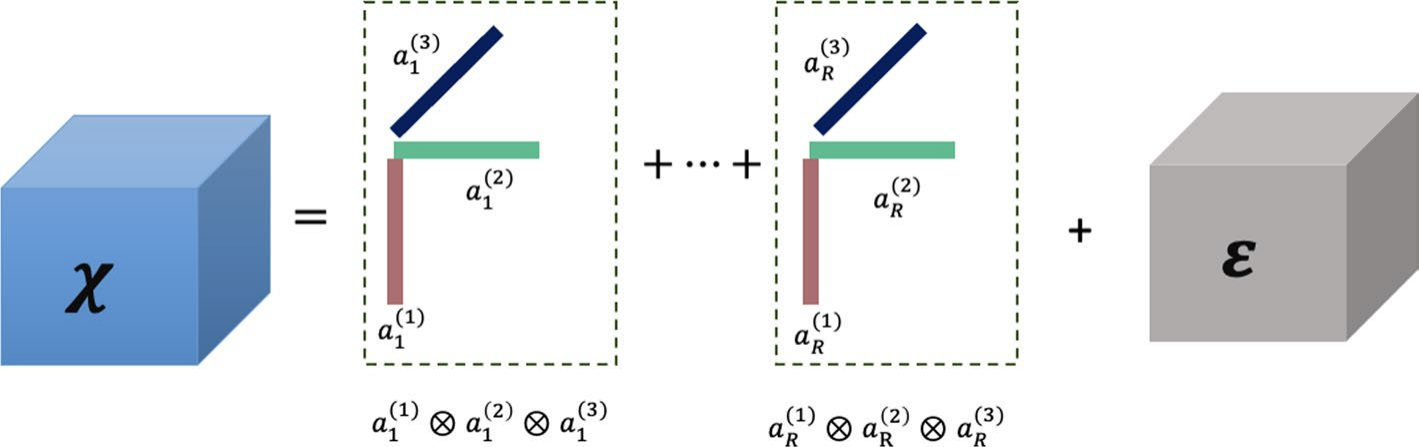
Low rank *R* approximation to a 3-way tensor ${\mathcal {X}}$ using CP decomposition

$A^{(i)}=[a^{(i)}_{1} \ldots a^{(i)}_{R} ] \in {\mathbb {R}}^{I_i \times R}, 1\le i \le N$ is the *latent* *factor matrix* along the $i-$th mode, and ${\mathcal {E}} \in {\mathbb {R}}^{I_1 \times I_2 \times \ldots \times I_N}$. The factorization given in Eq. (2) is often referred to as the CP (Candecomp/Parafac) decomposition. Figure 1 illustrates the CP decomposition of a 3-way tensor. The approximation can be concisely expressed as $\mathcal {\tilde{X}}=[[A^{(1)},A^{(2)},\ldots,A^{(N)}]].$ In this paper, we impose a non-negativity constraint on factors to improve their interpretability. The primary method for solving Eq. (2) involves using the maximum likelihood estimation (MLE) approach, which entails minimizing the following error:

3 $$\begin{aligned} \min _{A^{(1)},A^{(2)},\ldots,A^{(N)}} || {\mathcal {X}} - \mathcal {\tilde{X}}||_{F}. \end{aligned}$$

Iterative algorithms such as multiplicative updates, alternating least squares, and gradient descent are commonly utilized for Eq. (3) [10, 19, 21]. The MLE approach often assumes Gaussian noise [10, 19].

## Materials and methods

### Bayesian poisson tensor factorization

Traditional tensor factorization methods using MLE are unstable when applied to zero-inflated count data [11]. Bayesian Poisson Tensor Factorization (BPTF) extends the Poisson Matrix Factorization method to higher dimensions and utilizes Bayesian inference to obtain a point estimate and offers benefits such as uncertainty quantification, realistic noise assumptions, and principled inclusion of prior information [12–14, 22]. This section presents a general framework for BPTF with Variational Inference (VI) for high-dimensional count data.

Let ${\mathcal {X}} \in {\mathbb {R}}^{I_1 \times I_2 \times \ldots \times I_N}$ be the observed count data drawn from the Poisson distribution and with the CP decomposition as given in Eq. (2). Let $I=i_1i_2\ldots i_N \in {\overline{I}}=\{ i_1i_2 \ldots i_N~:~1 \le i_j \le I_j,~1\le j\le N\}$, then

4 $$\begin{aligned} \mathcal{X}_I \approx Poisson (\lambda _{I})~~~\text {where}~ {\mathcal {X}}_{I} \approx \mathcal {\tilde{X}}_{I}= \sum _{r=1}^{R} a^{(1)}_{i_{1}r} a^{(2)}_{i_{2}r} \ldots a^{(N)}_{i_{N}r} \approx \lambda _I. \end{aligned}$$

BPTF uses Gamma priors to regularize the estimation of the latent factors [23–25]. The Gamma distribution, which is characterized by a shape parameter $\alpha >0$ and a rate parameter $\alpha \beta > 0$, is employed as a sparsity-inducing prior [12, 13, 23]. Then for each $a^{(k)}_{jr}$ in Eq. (4), we have:

5 $$\begin{aligned} a^{(k)}_{jr} \approx Gamma(\alpha , \alpha \beta ^{(k)}),~1\le k \le N, \end{aligned}$$

with the expectation $E[a^{(k)}_{jr}]=\frac{1}{\beta ^{(k)}}$ and $Var[a^{(k)}_{jr}]=\frac{1}{\alpha {\beta ^{(k)}}^2}$. The posterior distribution given by $P(A^{(1)}, A^{(2)}, \ldots , A^{(N)}~|~{\mathcal {X}}, {\mathcal {H}})$ is intractable due to the inability to compute the evidence, given a model hyperparameter set ${\mathcal {H}} = \{\alpha,\beta^{(1)},\beta^{(2)},\ldots,\beta^{(N)}\}$ [25]. BPTF uses VI and assumes a variational family of distributions ${\mathcal {Q}}_V={\mathcal {Q}}(A^{(1)},A^{(2)},\ldots,A^{(N)}; V^{(1)},\ldots,V^{(N)})$ which is indexed by a set of variational parameters $V^{(k)}, 1\le k \le N$ [25, 26]. We employ a fully-factorized mean-field approximation assuming that $\mathcal {Q_V}(A^{(1)},A^{(2)},\ldots , A^{(N)})= \prod _{k=1}^{N} \mathcal {Q_V}(A^{(k)}; V^{(k)}),$ where ${\mathcal {Q}}(a^{(k)}_{jr}; V^{(k)}_{jr}) = Gamma(a^{(k)}_{jr}; \gamma ^{(k)}_{jr}, \delta ^{(k)}_{jr}), 1\le k \le N.$ The variational family *Q* used here is similar to the one employed in Bayesian Poisson Matrix Factorization [23, 27, 28]. BPTF fits variational parameters by minimizing the Kullback–Leibler (KL) divergence between the true posterior distribution and $\mathcal {Q_V}$, which is equal to maximizing the evidence lower bound (ELBO) [12, 25, 26]:

6 $$\begin{aligned} ELBO(V)= E_{Q_V}\big [log\big (P({\mathcal {X}}, A^{(1)},A^{(2)},\ldots , A^{(N)}~|~{\mathcal {H}})\big )\big]+ H(Q_V), \end{aligned}$$

where $H(Q_V)$ is the entropy for $Q_V.$ Coordinate ascent algorithms are commonly used to maximize the ELBO by iteratively optimizing each variational parameter while fixing the others until convergence, monitored by the relative change in the ELBO [25, 26]. From Eq. (4), we have the total $n= \sum _{I \in {\overline{I}}} {\mathcal {X}}_{I} \approx ~ Poisson(\Lambda )$ where $\Lambda =\sum _{I \in {\overline{I}}} \lambda _I.$ We can use the Poisson-Multinomial connection to express ${\mathcal {X}}$ given *n* as $Multinomial(n, \pi )$ where $(\pi )_{I}= \frac{\lambda _I}{\Lambda }$, and update variational parameters using this auxiliary distribution [13, 23, 25]:

7 $$\begin{aligned} \gamma^{(k)}_{jr}= & {} \alpha + \sum _{\begin{array}{c} i_1 i_2 \ldots i_N~\in~{\overline{I}} \\ i_k=j \end{array}} {\mathcal {X}}_{i_{1}i_{2}\ldots i_{n}} \frac{{\mathbb {G}}_{Q_V} \big [\prod _{s=1}^{N} a^{(s)}_{i_sr}\big ]}{\sum _{t=1}^{R} {\mathbb {G}}_{Q_V}\big [\prod _{s=1}^{N} a^{(s)}_{i_st}\big ]}, \end{aligned}$$

8 $$\begin{aligned} \delta ^{(k)}_{jr}= & {} \alpha \beta ^{(k)} + \sum _{i_1 i_2 \ldots i_N~\in~{\overline{I}}} E_{Q_V} \big [ \prod _{ 1\le s \ne k \le N}a^{(s)}_{i_sr}~\big ], \end{aligned}$$

where $E_{Q_V}[.]$ and ${\mathbb {G}}_{Q_V}=exp\big(E_{Q_V}\big[log(.)\big]\big)$ denote arithmetic and geometric expectations. Since $Q_V$ is fully factorized, the expectations in Equations (7) and (8) can be expressed as a product of individual expectations [25]. Specifically, for $a^{(s)}_{i_sr},$

9 $$\begin{aligned} E_{Q_V}[a^{(s)}_{i_{s}r}]=\frac{\gamma ^{(s)}_{{i_s}r} }{\delta ^{(s)}_{{i_s}r}}~~\text {and}~~{\mathbb {G}}_{Q_V}[a^{(s)}_{{i_s}r}]=\frac{exp\big(\Psi (\gamma ^{(s)}_{{i_s}r} )\big)}{\delta ^{(s)}_{{i_s}r}}, \end{aligned}$$

where $\Psi$ is the digamma function (logarithmic derivative of the gamma function). An empirical Bayes approach can be used to update the hyperparameters $\beta ^{(k)}, 1\le k \le N$, in conjunction with the variational parameters [23, 25]:

10 $$\begin{aligned} \beta ^{(k)}= \big ( \sum _{j=1}^{I_k} \sum _{r=1}^{R} {\mathbb {E}}_{Q_V}[a^{(k)}_{jr}]\big )^{-1}. \end{aligned}$$

The variational inference algorithm for BPTF is fully specified by the set of update equations Equations (7), (8), and (10).

### Zero-inflated poisson tensor factorization (ZIPTF)

Poisson models may not always be sufficient to model count data with excess zeros, and zero-inflated models can often provide a better fit [16, 17]. The Zero-Inflated Poisson (ZIP) model assumes that the counts in the tensor ${\mathcal {X}}$ can be modeled as a mixture of a point mass at zero and a Poisson distribution with parameter $\lambda$. Let ${\mathcal {X}}$ be a count data in ${\mathbb {R}}^{I_1 \times I_2 \times \ldots \times I_N}.$ We define the index set ${\overline{I}}$ as the collection of all possible indices, i.e., ${\overline{I}}=\{ i_1i_2 \ldots i_N~:~1\le i_j \le I_j,~1\le j\le N\}.$ We say ${\mathcal {X}}$ has Zero-inflated Poisson (ZIP) distribution if for every $I \in {\overline{I}}:$

11 $$\begin{aligned} P({\mathcal {X}}_{I}=x_{I})= p_I {\mathbb {1}_{x_{I}=0}} + (1-p_I) \frac{e^{-\lambda } \lambda ^{x_{I}} }{x_{I}!}, \end{aligned}$$

where the outcome variable $x_I$ has non-negative integer values, $\lambda _I$ is the expected Poisson count, and $p_I$ is the *probability of extra zeros* [17]. As an abbreviation, we write it as ${\mathcal {X}}_I \sim ZIP (\lambda _I, p_I).$ The ZIP can be considered as the *product* of a Poisson random variable ${\mathcal {Y}}_{I} \sim Poisson(\lambda _I)$ and an independent Bernoulli variable $\Phi _I \sim Bernoulli(p_I)$ [15]. The Bernoulli variable $\Phi _I$ takes the value of 1 when ${\mathcal {X}}_{I}$ is equal to 0, due to the Bernoulli component, and takes the value of 0 otherwise.

We consider the low rank $R \ge 1$ decomposition of the zero-inflated count tensor ${\mathcal {X}}:$

12 $$\begin{aligned} {\mathcal {X}} \approx {\mathcal {\tilde{X}}=} \sum _{r=1}^{R} a^{(1)}_{r}\otimes a^{(2)}_{r} \otimes \dots \otimes a^{(N)}_{r}. \end{aligned}$$

Hence, for $I=i_1 i_2\ldots i_N$, the reconstruction $\sum _{r=1}^{R} a^{(1)}_{i_{1}r} a^{(2)}_{i_{2}r} \ldots a^{(N)}_{i_{N}r}$ can be interpreted as the mean of the distribution from which the observed count ${\mathcal {X}}_{I}$ is assumed to be sampled. Then we have:

13 $$\begin{aligned} {\mathcal {X}}_{I} \sim ZIP (\lambda _I= \sum _{r=1}^{R} a^{(1)}_{i_1r} a^{(2)}_{i_2r} \ldots a^{(N)}_{i_{N}r}, p_{I}). \end{aligned}$$

### Variational inference for ZIPTF

For given position $I=i_1 i_2 \ldots i_N,$ we consider the rank *R* decomposition in Eq. (13). In Bayesian Poisson factorizations, the Gamma distribution is utilized as a prior to induce sparsity, and it is assumed that each latent factor matrix $A^{(k)} = [a^{(k)}_{1}\ldots a^{(k)}_{R}] \in {\mathbb {R}}_{+}^{I_k \times R},~1 \le k \le N$, follows a Gamma distribution [13, 23]. Therefore, for each $a^{(k)}_{jr}$ in Eq. (13), we have:

14 $$\begin{aligned} a^{(k)}_{jr} \sim Gamma(\alpha ^{(k)}, \beta ^{(k)}), \quad 1\le k \le N, \end{aligned}$$

where $\alpha ^{(k)} >0$ and $\beta ^{(k)}> 0$ represent the shape and rate parameters of the distribution, with the expectation $E[a^{(k)}_{jr}]=\frac{\alpha ^{(k)} }{\beta ^{(k)}}$ and $Var[a^{(k)}_{jr}]=\frac{\alpha ^{(k)}}{{\beta ^{(k)}}^2}$. Additionally, for ZIP models a latent variable $\xi$ is introduced to capture the hidden state of the probability of extra zeros which specify $\Phi \sim Bernoulli (p_I)$ [16, 25]. Let *S*(.) denote the *logistic sigmoid* function, given by $S(x)=\frac{1}{1 + e^{-x}}$, then:

15 $$\begin{aligned} \xi = S(\zeta )~~\text {where}~~\zeta \sim Normal(\mu , \sigma ). \end{aligned}$$

Let $Z= \{A^{(1)}, A^{(2)}, \ldots , A^{(N)}, \Phi \},$ consider the posterior distribution $P(Z~|~{\mathcal {X}}, {\mathcal {H}}),$ given a model hyperparameter set ${\mathcal {H}} = \{\alpha ^{(1)}, \beta ^{(1)}, \alpha ^{(2)}, \ldots , \beta ^{(2)}, \ldots , \alpha ^{(N)}, \beta ^{(N)}, \mu , \sigma \}.$

Variational inference approximates the true posterior distribution using a family of probability distributions ${\mathcal {Q}}$ over hidden variables [25]. This family of distributions is characterized by free parameters, and the key assumption is that each latent variable is independently distributed given these parameters. We assume a variational family of distributions ${\mathcal {Q}}$ indexed by a set of variational parameters $V=\{\gamma ^{(1)}, \delta ^{(1)},\gamma ^{(2)}, \delta ^{(2)}, \dots , \gamma ^{(N)}, \delta ^{(N)}, {\overline{\mu }}, {\overline{\sigma }}\}$ where $(\gamma ^{(k)}, \delta ^{(k)})$ are variational shape and rate parameters of the Gamma distribution for the latent factor along the $k-$th mode, and $({\overline{\mu }}, {\overline{\sigma }})$ are the variational parameters for $\zeta$. We use a fully factorized mean-field approximation [25] and the variational distribution factors as the following:

16 $$\begin{aligned} {\mathcal {Q}} (A^{(1)},A^{(2)},\ldots , A^{(N)}, \Phi )= {\mathcal {Q}}( \Phi ; {\overline{\mu }}, {\overline{\sigma }}) \prod _{k=1}^{N} {\mathcal {Q}}(A^{(k)};\gamma ^{(k)}, \delta ^{(k)}), \end{aligned}$$

where $a^{(k)}_{jr} \sim Gamma( \gamma ^{(k)}_{jr},\delta ^{(k)}_{jr})$ and $\Phi _{I} \sim Bernoulli\big(S(\zeta )\big)~\text {for}~\zeta \sim Normal({\overline{\mu }}, {\overline{\sigma }})$. The goal is to choose a member $q^{*}$ of the family of variational distributions which minimizes the KL divergence of the exact posterior from ${\mathcal {Q}}$:

17 $$\begin{aligned} q^{*}(Z) = \arg \min _{q(Z)~\in~{\mathcal {Q}}} D_{KL}\big( q(Z)~ \big \Vert ~ P(Z~|~{\mathcal {X}}, {\mathcal {H}})\big) . \end{aligned}$$

Upon examining the KL divergence, we encounter a significant challenge: it involves the true posterior distribution $P(Z~|~{\mathcal {X}}, {\mathcal {H}})$, which is not known. Nevertheless, we can rewrite the KL divergence as follows:

18 $$\begin{aligned} D_{KL} \big( q\big(Z~\Vert~P(Z~|~{\mathcal {X}}, {\mathcal {H}})\big) \big)= & {} \int q(Z) \log \left( \frac{q(Z) }{P(Z~|~{\mathcal {X}}, {\mathcal {H}} )} \right) dZ~~~~~~~~ \end{aligned}$$

19 $$\begin{aligned}= & {} \int q(Z) \log \left( \frac{q(Z) P({\mathcal {X}}, {\mathcal {H}} )}{P(Z,{\mathcal {X}}, {\mathcal {H}})} \right) dZ \end{aligned}$$

20 $$\begin{aligned}= & {} \log \big( P({\mathcal {X}}, {\mathcal {H}} )\big) \int q (Z) dZ- \int q (Z) \log \left( \frac{P(Z,{\mathcal {X}}, {\mathcal {H}} )}{ q(Z)}\right) dZ~~~~~~~~ \end{aligned}$$

21 $$\begin{aligned}= & {} \log \big( P({\mathcal {X}}, {\mathcal {H}} )\big) - \int q (Z) \log \left( \frac{P(Z,{\mathcal {X}}, {\mathcal {H}} )}{ q(Z)}\right) dZ. \end{aligned}$$

The second term in  Eq. (21) is called Evidence Lower Bound (ELBO). We know that the KL divergence is non-negative, therefore, $\log \big( P({\mathcal {X}}, {\mathcal {H}} )\big) \ge ~\text {ELBO}\big(q(Z)\big)=\int q (Z) \log \left( \frac{P(Z,{\mathcal {X}}, {\mathcal {H}}) }{ q (Z)}\right) dZ.$

22 $$\begin{aligned} \text {ELBO}\big(q(Z)\big) &= \int q(Z) \log \big( P(Z,{\mathcal {X}}, {\mathcal {H}} )\big) dZ - \int q(Z) \log \big( q(Z)\big) dZ \end{aligned}$$

23 $$\begin{aligned} &= \mathbb{E}_{q(Z)} \big[\log \big( P({\mathcal {X}},Z, {\mathcal {H}})\big) \big]-\mathbb{E}_{q(Z)}\big[\log q(Z)\big]. \end{aligned}$$

The evidence lower bound serves as a transformative tool that converts intractable inference problems into optimization problems that can be tackled using gradient-based methods [25].

Coordinate ascent algorithms are frequently employed in maximizing the evidence lower bound [16, 25]. However, these algorithms require tedious gradient calculations and may not scale well for very large datasets [29, 30]. Closed-form coordinate-ascent updates are applicable to conditionally conjugate exponential family models, but they necessitate analytic computation of various expectations for each new model [29, 30].

Stochastic Variational Inference (SVI) [29] offers a more efficient algorithm by incorporating stochastic optimization [31]. This technique involves utilizing noisy estimates of the gradient of the objective function. To maximize the ELBO, we employ a stochastic optimization algorithm known as the *Black Box Inference Algorithm* [30]. This algorithm operates by stochastically optimizing the variational objective using Monte Carlo samples from the variational distribution to compute the noisy gradient (see Sect. 2, [30] for details). By doing so, it effectively alleviates the burden of analytic computations and provides a more efficient approach to ELBO maximization.

**Fig. 2:**
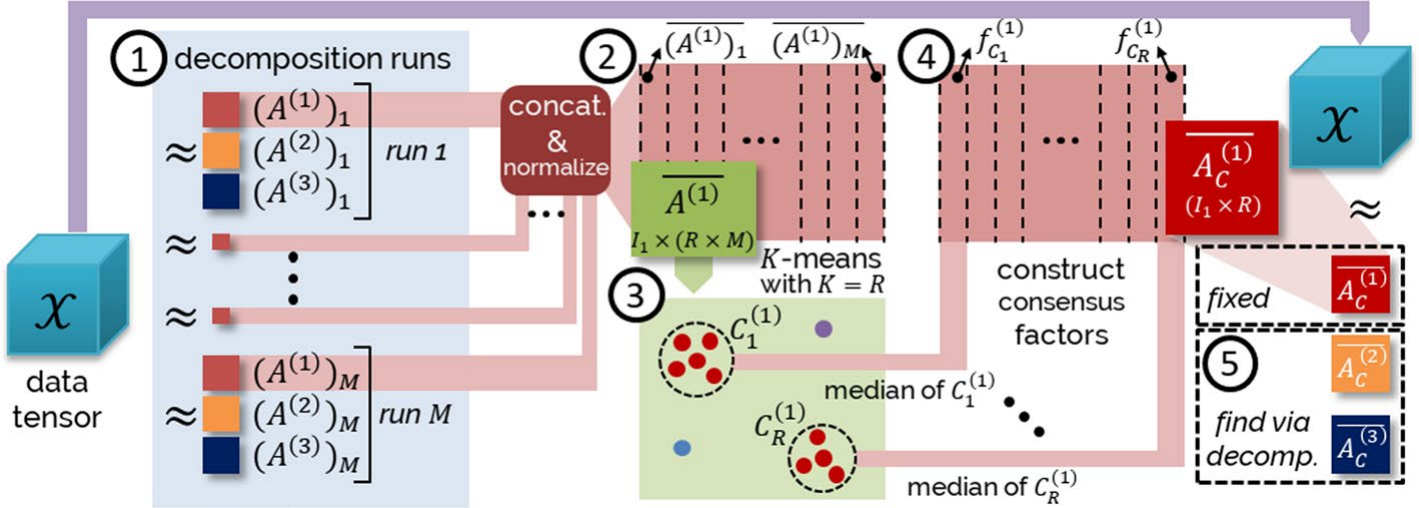
Overview of the consensus meta-analysis approach discussed in Section  Generic consensus-based tensor factorization for the 3-way tensor ${\mathcal {X}}$

### Generic consensus-based tensor factorization

Selecting the number of components in tensor factorization is challenging [10, 21]. The dependence on initial guesses for latent factors can lead to substantially different factor sets across repeated runs, making it difficult to interpret the results [10, 19, 21]. Traditional approaches involves selecting the minimum *R* in Eq. (12) that surpasses a predetermined threshold for the *explained variance of the approximation*, defined as follows:

24 $$\begin{aligned} \text {explained variance}=1- \frac{||{\mathcal {X}}- \mathcal {\tilde{X}}||_{F}}{||{\mathcal {X}}||_{F}}. \end{aligned}$$

As the rank *R* increases, explained variance also increases, owing to the increased flexibility to capture the intricacies of the original tensor ${\mathcal {X}}$. However, depending solely on explained variance for selecting the rank may not always yield the best outcomes. Higher ranks can lead to overfitting, capturing noise instead of meaningful data patterns, thus inflating explained variance. Additionally, the increased computational complexity of higher ranks may not justify the marginal improvement in explained variance. Moreover, interpreting factors from high-rank tensor decompositions becomes more challenging, potentially obscuring the underlying data structure. Our goal is not solely to improve the explained variance, but also to ensure the interpretability and stability of the factors. We explore additional metrics to assess the stability of factors, namely the cophenetic correlation and silhouette score; further details are provided below.

We generalize the consensus meta-analysis approach, which has been previously used for matrix factorization [20], and include novel techniques to enhance the stability. The overview of the proposed pipeline is depicted in Fig. 2. In the remainder of this section, we will refer to the steps $\textcircled {1}$-$\textcircled {5}$ given in the figure.

Running a generic rank *R* factorization given in Eq. (12) for  ${\mathcal {X}} \in {\mathbb {R}}^{I_1 \times I_2 \times \ldots \times I_N}$ with *M* different random seeds yields the sets of non-negative factor matrices $\{(A^{(1)})_{m}, (A^{(2)})_{m},\ldots , (A^{(N)})_{m} \}, ~1\le m \le M$ (Step $\textcircled {1}$). For a chosen modality $k~(1\le k\le N$), we can aggregate and normalize the factor matrices from independent runs (Step $\textcircled {2}$):

25 $$\begin{aligned} \overline{A^{(k)}}=\Big [ \frac{(A^{(k)})_{1}}{||(A^{(k)})_{1}||_{F}}~\frac{(A^{(k)})_{2}}{||(A^{(k)})_{2}||_{F}}~\ldots \frac{(A^{(k)})_{M}}{||(A^{(k)})_{M}||_{F}} \Big ] \in I_k \times (R \times M). \end{aligned}$$

The *cophenetic correlation coefficient*, a commonly employed metric for selecting ranks in matrix factorizations [32], assumes a one-to-one mapping between features and factors, primarily based on maximum loadings and evaluating the level of stability of inferred latent signals across different runs. While this approach is valuable, it’s important to acknowledge that this assumption may encounter challenges in scenarios where a feature contributes significantly to multiple factors. Despite this limitation, the cophenetic correlation coefficient remains a valuable tool for rank selection in many cases.

Our method for selecting the rank and evaluating factorization stability involves clustering column factors of aggregated matrices and fixing the initial guess to ensure reliability. Initially, we perform K-means clustering [33] on the columns of the aggregated factor matrix $\overline{A^{(k)}}$ with $K=R$ (Step $\textcircled {3}$). The resulting cluster sets are given as $C^{(k)} _{i}= \{ \text {columns of}~\overline{A^{(k)}}~\text {assigned to cluster}~ i\}, 1\le i \le R.$ The Local Outlier Factor algorithm [34] is used to remove outliers by considering the local density deviation of a data point compared to its neighbors. We evaluate the *goodness* of the clustering with the *silhouette coefficient* [35], computed as (b-a)/max(a,b), where a is the average intra-cluster distance, and b is the average inter-cluster distance. The silhouette coefficient ranges from -1 to 1, with higher values indicating more coherence. After clustering, we obtain the consensus factors $f^{(k)}_{C_i}$, where $1\le i\le R$, by computing the median value of the factors in each cluster (Step $\textcircled {4}$) and form the consensus matrix:

26 $$\begin{aligned} \overline{A^{(k)}}_{C}=[ f^{(k)}_{C_1} f^{(k)}_{C_2} \ldots f^{(k)}_{C_{R}} ] \in {\mathbb {R}}^{I_k \times R}, 1\le k \le N. \end{aligned}$$

We perform the decomposition using $\overline{A^{(k)}}_{C}$ as the fixed initial guess for the *k*-th modality to obtain the final factor matrices (Step $\textcircled {5}$).

Notice that if ZIPTF is employed as the factorization method in Step $\textcircled {1}$ described above, we refer to the resulting factorization as C-ZIPTF.

In summary, when selecting the rank *R*, we ensure that the explained variance surpasses a predefined threshold of 0.9. Additionally, we evaluate the cophenetic correlation across various ranks to validate our selection, aiming for a score higher than 0.9. Next, at the candidate rank, we assess the silhouette score to determine if the optimal number of clusters of latent factors from multiple runs of the factorization aligns with the chosen rank.

It is important to emphasize that achieving the mathematically “ideal” rank does not ensure optimal performance in capturing biologically relevant signals. To ensure that biological signals are adequately preserved, it is essential to complement mathematical optimization with domain knowledge, exploratory analysis, and validation against biological criteria.

When assessing the sensitivity of parameter *R*, it’s important to consider the implications from a biological standpoint. Opting for a rank that is too low may lead to loss of important biological information, while excessively high rank may result in overfitting. It’s crucial to recognize that factorizing at a given rank still captures the true signal but potentially at a different resolution. Lower ranks may capture higher-level shared signals, while higher ranks may reveal finer subgroups within these categories. For example, in a 2-way case with gene expression count data for different donors (donors $\times$ genes), factorizing it with $R=1$ would produce a gene latent factor based on average expression of genes. Increasing to higher ranks may reveal distinct groups, such as disease/healthy, while even higher ranks may uncover finer subgroups within these categories. At some point, we would start capturing donor-specific signals. In Section  Identification of cell type identity and perturbation-specific programs using C-ZIPTF in single-cell RNA-seq data, we examine biological signals obtained through factorization at different ranks.

## Results

Here we present results showing the superior performance of C-ZIPTF across multiple evaluation metrics. We implemented C-ZIPTF in Python, using the probabilistic programming language Pyro [36]. Our presentation in Section  Bayesian poisson tensor factorization focused on Bayesian tensor factorization frameworks that utilize Poisson and Zero-Inflated Poisson based models. However, our implementation is designed to be more versatile and can accommodate different types of noise models. We conduct four different evaluations to assess the performance of our proposed method. First, we compare ZIPTF with alternative tensor factorization methods on simulated tensors with known factors and ZIP noise and evaluate the benefits of using a ZIP model and the inclusion of the consensus approach (Section  Synthetic tensor experiment).

After observing the superior performance of ZIPTF and C-ZIPT on zero-inflated simulated count tensors compared to traditional tensor factorization methods, we applied our approach to genomics datasets to evaluate its effectiveness in dimension reduction and capturing gene expression patterns as a factor analysis method. Factor analysis techniques like Independent Component Analysis, Linear Discriminant Analysis, Non-negative Matrix Factorization (NMF) [37], and Principal Component Analysis (PCA) [38] are frequently employed for dimensionality reduction tasks in genomic data analysis [39–42]. The algorithmic variability of the first three methods necessitates a consensus meta-analysis for robustness. Consensus NMF (CNMF) has emerged as the top-performing model across various simulation settings [20], outperforming PCA as well. Consequently, we conducted a comparative analysis that included both NMF and CNMF as matrix-based methods. Furthermore, we integrated deep learning-based Amortized Latent Dirichlet Allocation (LDA) into our comparison. LDA, an unsupervised learning technique, assumes a generative model, where latent topics generate collections of elements [43]. Amortized LDA is implemented using autoencoding variational Bayes in which a fully-connected neural network is used as the encoder. When applied to scRNA-seq data, topics correspond to gene modules, while each cell corresponds to a collection of Unique Molecular Identifier (UMI) counts.

In Section  Synthetic single-cell RNA-Seq data analysis, we evaluate the performance of our method on simulated multi-donor single-cell RNA sequencing data and compare it with other matrix and tensor factorization methods, as discussed above, at the task of recovering identity and activity gene expression programs (GEPs). In Section Identification of cell type identity and perturbation-specific programs using C-ZIPTF in single-cell RNA-seq data, we demonstrate the ability of our method to capture biologically meaningful gene expression programs by applying it to a real multi-sample multi-condition scRNA-seq dataset of immune cells stimulated with interferon beta. Finally, we apply C-ZIPTF to a real multi-sample scRNA-seq data obtained from patients with systemic lupus erythematosus (including those with managed and active flare) and healthy groups (Section C-ZIPTF enables unsupervised discovery of disease subgroups and multicellular gene expression programs in the peripheral blood of patients with systemic lupus erythematosus). We showcase C-ZIPTF’s efficacy in capturing condition-specific GEPs, revealing nuanced patterns that highlight intra-group heterogeneity that are typically missed by traditional supervised methods such as differential gene expression (DGE) analysis.

**Fig. 3:**
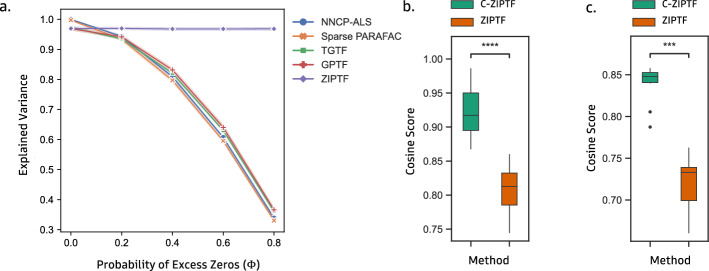
ZIPTF compared to alternative factorization methods on a synthetic tensor with known factors with predetermined rank and ZIP noise, and stability comparison between ZIPTF and C-ZIPTF: **a** we calculated the explained variance of ZIPTF and alternative methods for different levels of extra zeros, **b** cosine similarity between factors obtained on repeat runs for ZIPTF and C-ZIPTF, **c** cosine similarity between inferred factors and original factors for ZIPT and C-ZIPTF

### Synthetic tensor experiment

To evaluate the performance of C-ZIPTF on synthetic data, we generate tensors using known factors and Poisson noise with varying degrees of zero inflation and measure the accuracy of different methods at recovering the original factors. To generate a tensor $\mathcal {T'}\in {\mathbb {R}}^{I\times J \times K}$, we first create three factor matrices $A\in {\mathbb {R}}_{+}^{I\times R}$, $B\in {\mathbb {R}}_{+}^{J\times R}$, and $C\in {\mathbb {R}}_{+}^{K\times R}$, with elements drawn from a Gamma distribution with shape $\alpha =3$ and rate $\beta =0.3$, where *R* is the desired true rank. We then construct a tensor ${\mathcal {T}}$ by taking the sum of the outer product of the corresponding columns of the matrices, i.e., ${\mathcal {T}} = \left[\left[A,B,C\right]\right]$. Finally, we generate the tensors ${\mathcal {T}}'$ by sampling from a ZIP distribution with mean ${\mathcal {T}}$ and a given probability of extra zeros, denoted by $\Phi$ in Section 3.2.

#### Zero-inflated Poisson model results in higher explained variance

For the first experiment, we ran ZIPTF without consensus aggregation to evaluate the advantages of using the ZIP model alone. In this comparative analysis, we initially compare our method with the traditional non-Bayesian tensor method, Non-Negative CP decomposition via Alternating-Least Squares (NNCP-ALS) along with Sparse PARAFAC, which efficiently handles sparsity with L2 regularization [10, 11, 44]. Afterward, we transition to Bayesian methods, starting with the Bayesian Tensor Factorization using a Truncated Gaussian Model (TGTF) [12]. Subsequently, we run Bayesian Tensor Factorization with Gamma Poisson model (GPTF) [13] which assumes Poisson distribution for the data and Gamma prior on the latent factors. We conducted 20 trials, generating a new simulated tensor ${\mathcal {T}}'$ of shape $10 \times 20 \times 300$ and rank 9 each time and running each factorization method on the tensor for a fixed maximum number of iterations ($max\_iter = 1000$). We evaluated the performance of the methods using the explained variance (24) of the approximation generated by each factorization. ZIPTF consistently outperformed all the methods included in the comparison Fig. 3a. At a zero probability of excess zeros, all four methods showed similar and nearly perfect explained variance. However, as the excess zero level increased, the performance of the other methods deteriorated rapidly. At the highest probability of excess zeros simulated, $\Phi = 0.8$, the average explained variance of the ZIPTF approximation was 0.974, with a 95% confidence interval (CI) [0.962, 0.987], about $2.4\times$ better than the second highest explained variance of 0.338, 95% CI [0.334, 0.342] achieved by the Gamma Poisson model. We also note that the difference in explained variance between NNCP-ALS and the Bayesian methods other than ZIPTF is minimal compared to the difference to ZIPTF. This indicates that the superiority of ZIPTF arises from using the appropriate noise model.

#### Consensus aggregation leads to more consistent factorization

After demonstrating ZIPTF’s superior performance in modeling zero-inflated count data, we examine the benefits of consensus aggregation. We generate tensors of shape $40 \times 20 \times 2000$ and rank 9 with known factors and Zero-Inflated Poisson noise as described above and evaluate the recovered factors by running ZIPTF with and without consensus aggregation. For this experiment, we fix the probability of excess zeros $\Phi = 0.6$. We compare the internal consistency of factors obtained from multiple runs of the decompositions. For our simulated tensor ${\mathcal {T}}',$ assume that we have two rank R approximations [[*A*, *B*, *C*]] and [[*D*, *E*, *F*]] corresponding to different randomly initialized runs. To measure the similarity between factorizations, we calculate:

27 $$\begin{aligned} \textit{cosine score}\big([[A,B,C]], [[D,E,F]]\big)= \frac{1}{R}\sum _{i=1}^{R} \max _{1\le j \le R}~ \cos (a_i, d_j)\cos (b_i,e_j) \cos (c_i, f_j). \ \end{aligned}$$

We evaluate the similarity of factors recovered from 20 randomly initialized runs of both ZIPTF and C-ZIPTF using the *cosine score* given in Eq. (27). We observe that the factors recovered from C-ZIPTF are more consistent with one another compared to those recovered from ZIPTF, as shown in Fig. 3b. The consensus approach makes C-ZIPTF more robust, reducing the impact of the inherent stochasticity of the factorization process and resulting in a more stable set of factors.

#### Consensus aggregation leads to more accurate recovery of original factors

We assess the accuracy of both ZIPTF and C-ZIPTF in recovering the original factors used to create the tensor with $\Phi =0.6$. We perform 20 randomly initialized runs of each method and compare the recovered factors to the original factors using the *cosine score*. Figure 3c demonstrates that C-ZIPTF outperforms ZIPTF in recovering the original factors.

### Synthetic single-cell RNA-Seq data analysis

**Fig. 4:**
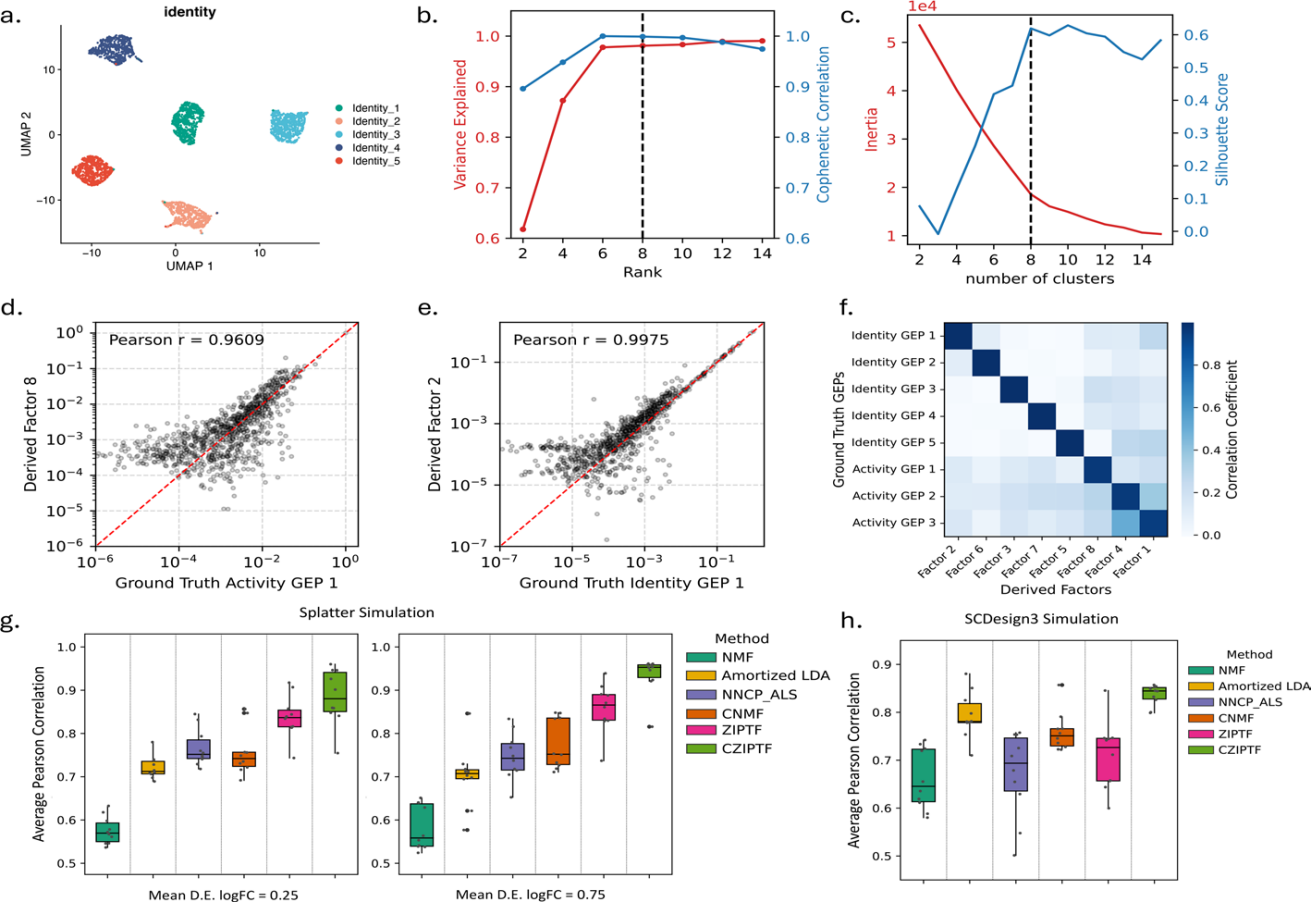
Recovering GEPs from synthetic single-cell RNA-seq data: **a** UMAP of cells simulated using the Splatter framework, colored by the dominant identity GEP expressed by each cell, **b** explained variance and gene mode cophenetic correlation of C-ZIPTF factorizations of the simulated data at different ranks, **c** Silhouette score and inertia of the K-means clustering of gene components resulting from 10 randomly initialized factorization at rank 8, **d, e** correlation between the ground truth identity (d) and activity (e) GEPs used in the simulation and the corresponding GEPs inferred by C-ZIPTF, **f** pairwise Pearson correlation between each of the eight latent factors in the gene mode obtained via C-ZIPTF factorization and the original GEPs, **g, h** the average Pearson correlation between the true GEPs used in the simulation and the inferred GEPs obtained from various factorization methods, results are presented for two different signal intensity levels (0.25 and 0.75), which are indicated by the mean log2 fold change (log2FC) of simulated differentially expressed genes, (g) presents results from simulation done using Splatter, while (h) illustrates results from simulations conducted with scDesign3

We test the performance of C-ZIPTF on simulated single-cell RNA sequencing data which is prone to zero inflation due to technical limitations that result in dropout events [4]. We used the Splatter simulation framework [45] which was adapted to Python in a previous study [20] to generate the synthetic scRNA-seq dataset. The simulation framework utilizes a Gamma-Poisson hierarchical model with hyperparameters estimated from real data. Technical dropouts are simulated by randomly replacing some of the simulated counts with zeros using a Bernoulli distribution. The complete details of the simulation framework and parameters used are provided in Section Implementation of ZIPTF and C-ZIPTF. The synthetic dataset consists of 3000 cells and 1000 genes from six donors, with five gene expression programs defining cell type identities and three gene expression programs defining donor-specific activity (Fig. 4a).

To apply C-ZIPTF to this data, we first construct the observed tensor by pseudobulking the cell-by-gene counts matrix. We cluster the cells to obtain tentative cell type groupings and generate pseudobulk counts by summing all the counts for each donor, cell type, and gene. This creates a tensor of shape $D \times C \times G$, where D, C, and G represent the number of donors, cell types, and genes, respectively. We then normalize the pseudobulk tensor to counts per million (CPM) and run C-ZIPTF on this tensor for a set of ranks ranging from 2 to 14, conducting 10 random initialized factorization at each rank. Since the simulated data has 5 identity GEPs and 3 activity GEPs we expect factorizations of rank 8 or more to be able to sufficiently represent the data. As anticipated, at rank 8, the factorization exhibited an explained variance and cophenetic correlation greater than 0.98 (Fig. 4b). The silhouette score for the K-means clustering of gene latent factors from the 10 randomly initialized factorizations at rank 8 also reached its peak at 0.61 with 8 clusters (Fig. 4c). These results demonstrate the utility of explained variance, cophenetic correlation and silhouette score in estimating the rank of the data.

To evaluate the accuracy of the C-ZIPTF in recovering the eight true GEPs, we computed the Pearson correlation [46] between each of the eight latent factors in the gene mode obtained via factorization and the original GEPs (Fig. 4d and e). As seen in Fig. 4f, there is a clear one to one relationship between the ground truth GEPs and the gene factors from C-ZIPTF. The Pearson correlation between the aligned pairs of GEP and gene factor (diagonal entries in Fig. 4f) was consistently high (mean = 0.98, SD $= 0.02$).

Next, we compared the performance of C-ZIPTF against various factorization methods, including ZIPTF, NMF, CNMF, Amortized LDA and NNCP-ALS, at the task of recovering the 8 GEPs embedded in the synthetic scRNA-seq dataset. For NMF and CNMF, the decomposition at rank 8 is performed with a maximum of 1000 iterations for convergence after normalizing the cell-by-gene count matrix to CPM. For the tensor-based approaches, we again construct the observed tensor by pseudobulking the cell-by-gene counts matrix as described above and apply tensor factorization methods with rank 8 and perform 1000 iterations for each method. For each method, we computed the Pearson correlation between each of the eight gene latent factors and the original GEPs. This correlation was used to establish a one-to-one alignment between the factors and the GEPs. We calculated the average Pearson correlation between each factor and its corresponding GEP as the overall accuracy score of the method. We ran ten trials of this analysis and report the results in Fig. 4g for two different levels of simulated intensity of activity GEPs (mean log2 fold change of differentially expressed (D.E.) genes (log2FC) $\in \{0.25, 0.5, 0.75 \}$). C-ZIPTF outperformed all the compared matrix and tensor factorization methods (Fig. 4g). At mean D.E. logFC of 0.75, C-ZIPTF generated factors with the highest average Pearson correlation to the simulated GEPs (mean Pearson $r = 0.93$; SD $= 0.04$). ZIPTF without consensus aggregation also performed better than the rest of the factorization methods (mean Pearson $r = 0.85$; SD $= 0.04$), indicating that both the use of the ZIP model and the consensus aggregation independently improve the accuracy of the method in recovering GEPs. The same pattern was observed at mean D.E. logFC of 0.25. C-ZIPTF recovered GEPs at a higher accuracy (mean Pearson $r = 0.89$; SD $= 0.05$) than all the other methods followed by ZIPTF (mean Pearson $r = 0.83$; SD $= 0.05$).

Finally, we evaluate the factorization methods on synthetic scRNA-seq data simulated using a different simulation framework, scDesign3 [47]. Importantly, instead of a ZIP distribution, scDesign3 was configured to use a Gaussian marginal distribution for genes to test the performance of C-ZIPTF under different underlying distributions. We simulate a dataset with six cell types and one condition effect with two discrete levels using a total of 8 GEPs. The final simulation resulted in a count matrix of 2,400 cells and 600 genes. We process this count matrix like above to generate a normalized matrix and a pseudobulk tensor and run the same set of factorization methods with rank 8. We again found that C-ZIPTF generated factors with the highest average Pearson correlation to the simulated GEPs (mean Pearson *r* = 0.84; SD $= 0.02$) (Fig. 4h). However, the factors generated by ZIPTF were not significantly more accurate than those generated by NMF and NNCP-ALS. This was expected due to the Gaussian marginal distribution used in the data simulation, which lacks zero inflation. The performance benefits of ZIPTF are generally proportional to the level of zero inflation in the data, and the absence of zero inflation minimizes the advantage of using ZIPTF as seen in Fig. 3a. Despite this, it is notable that ZIPTF’s performance does not deteriorate below that of standard factorization methods even when zero inflation is absent. This result underscores that while ZIPTF doesn’t offer significant advantages without zero inflation, it remains a robust method that doesn’t lose ground compared to standard approaches in scenarios where zero inflation is not present. Notably, the advantages gained from applying consensus aggregation are still retained even when the underlying distribution is different as can be seen by the fact that C-ZIPTF still outperforms the other methods.

### Identification of cell type identity and perturbation-specific programs using C-ZIPTF in single-cell RNA-seq data

**Fig. 5:**
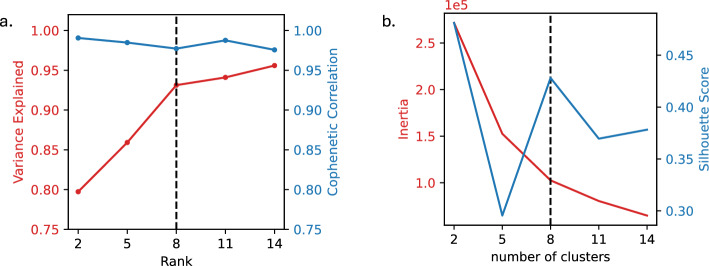
Metrics used in selecting the optimal rank for running C-ZIPTF on real single-cell RNA sequencing dataset [48] of immune cells stimulated with interferon beta (IFN-$\beta$): **a** explained variance and cophenetic correlation within a range of ranks from 2 to 14, **b** the K-means inertia and silhouette score were evaluated across varying numbers of clusters of gene latent factors, while keeping the rank fixed at 8

We applied C-ZIPTF to a real-world single-cell RNA sequencing dataset of peripheral blood mononuclear cells (PBMCs) from patients with Lupus, reported in [48]. The dataset has been deposited in the Gene Expression Omnibus under the accession number GSE96583. As described in [48], the dataset contains 29,065 cells from eight patients, which are divided into stimulated and control groups, with the former being treated with interferon beta (IFN-$\beta$), a cytokine that modulates the transcriptional profile of immune cells. As part of the preprocessing step, we filter out multiplets and cells without a cell type assignment. Additionally, we remove samples and cell types that constitute less than 2 percent of cells. After these filtering steps, the dataset contained 14 samples, 7 control and 7 stimulated, and 6 cell types: CD4 T cells, CD14+ monocytes, B cells, CD8 T cells, NK cells, and FCGR3A+ monocytes. In order to facilitate biological interpretability of factors and reduce noise in the tensor formed we removed genes that are either not provided with HGNC symbols [49], or had a total count of less than 50 across all cells. Finally, we create a pseudobulk tensor by summing up the raw counts for each cell type, sample, and gene. The resulting pseudobulk data tensor has dimensions S $\times$ C $\times$ G (14 $\times$ 6 $\times$ 9,276), where S, C and G denote the number of samples, cell types and genes respectively. We normalize the tensor such that each sample-cell type pair has a total of $10^6$ counts. We first determined the optimal rank for the data by running C-ZIPTF with a range of ranks from 2 to 14 and using 5 random initialization each time. Figure 5 illustrates several metrics that we examined to determine the best rank and cluster number, such as explained variance, gene cophenetic correlation, and silhouette score. We choose rank 8, given its high explained variance and strong cophenetic correlation ($>0.9$) as shown in Fig. 5a. We confirmed that the optimal number of clusters of gene latent factors obtained from multiple runs at rank 8 is indeed 8, reaching a peak for the silhouette score, as illustrated in Fig. 5b.

**Fig. 6:**
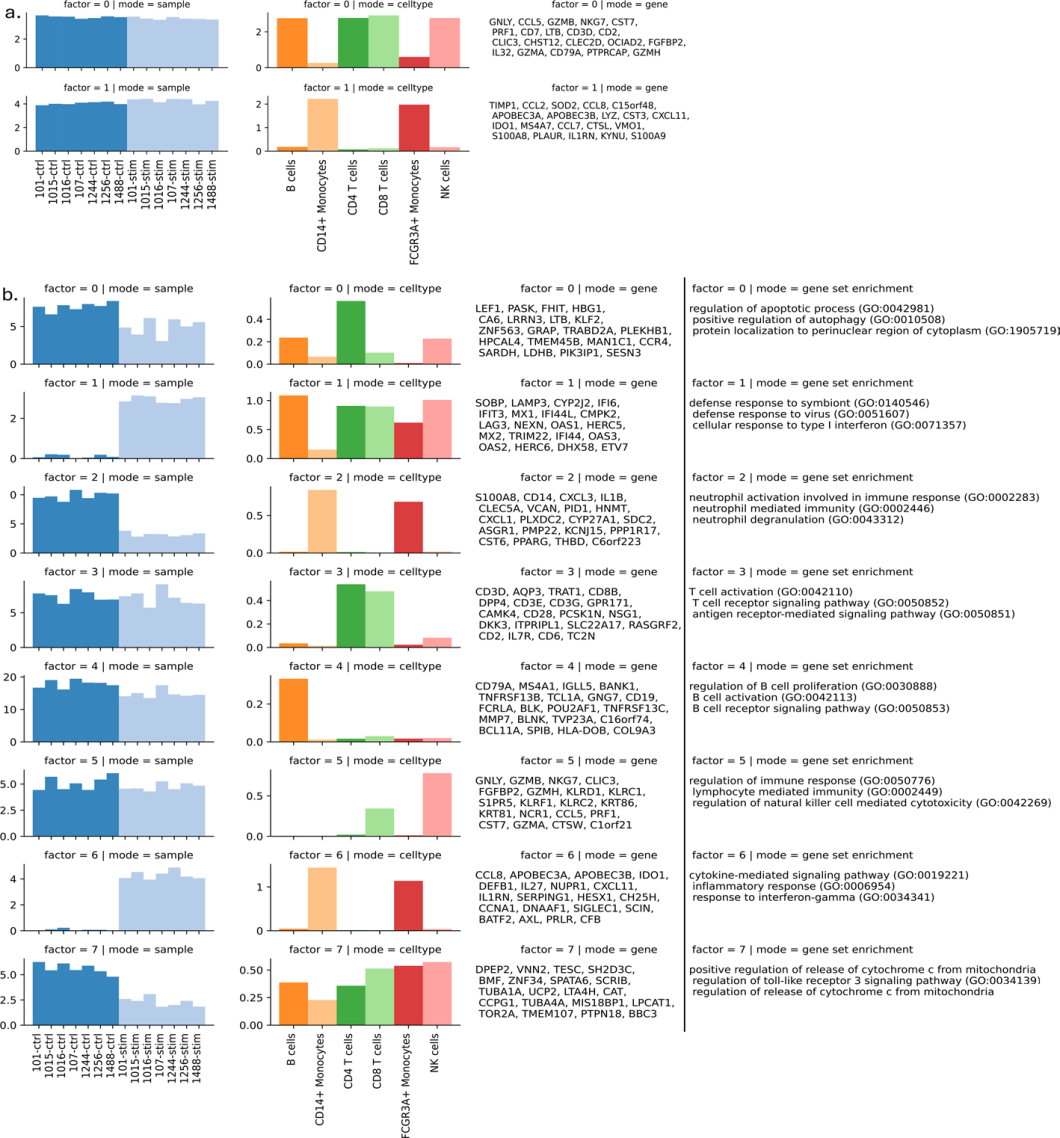
The full set of factors recovered by running C-ZIPTF on real single-cell RNA sequencing dataset [48] of immune cells stimulated with interferon beta (IFN-$\beta$): **a** rank$=2$, **b** rank=8. Each row represents a factor, and the first three columns display the three modes: sample, cell type, and gene. The $y-$axis in the sample and cell type modes represent the loading of the sample or cell type on that factor. The gene mode exhibits the top 20 genes associated with the factor. The last column provides the top 3 enriched terms obtained from a gene set enrichment analysis

As discussed in Section Generic consensus-based tensor factorization, different ranks can capture meaningful structures in the data at different resolutions. For example, at lower ranks such as at rank 2, as shown in Fig. 6a, C-ZIPTF captures very high-level structures in the data, such as lymphoid (B cells, CD4+ T cells, CD8+ T cells and NK cells) versus myeloid (CD14+ monocytes and FCGR3A+ monocytes) lineage. While at rank 8, C-ZIPTF successfully identifies high-resolution structures, including finer cell type and condition-specific gene expression programs, Fig. 6b.

Notably, factor 4 represents an identity GEP that remains active in all B cells, irrespective of the condition. The genes exhibiting the highest loadings for this factor are well-established B cell markers, such as *MS4A1*, *CD79A*, and *BANK1* [50]. Furthermore, we performed gene set enrichment analysis [51] of these factors using GSEApy [52] in Python. This analysis revealed enrichment pathways consistent with B cell characteristics, including B cell activation and the B cell receptor signaling pathway.

Conversely, factor 1 and factor 6 capture distinct gene expression programs that are specifically activated in IFN-$\beta$ stimulated samples. Factor 1 captures a cross-cell-type response to IFN-$\beta$ stimulation, whereas factor 6 represents a monocyte-specific response. These findings align with previous studies that have reported a monocyte-specific response to IFN-$\beta$ stimulation [53]. Furthermore, gene set enrichment analysis revealed enrichment in pathways such as the cellular response to type I interferon and inflammatory response, among others. For a comprehensive list of factors identified by C-ZIPTF and their associated gene expression programs, please refer to Fig. 6b.

### C-ZIPTF enables unsupervised discovery of disease subgroups and multicellular gene expression programs in the peripheral blood of patients with systemic lupus erythematosus

**Fig. 7:**
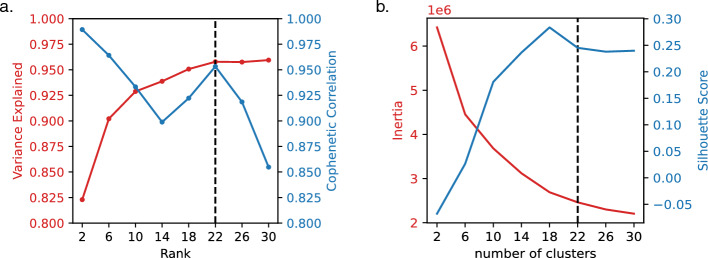
Metrics used in selecting the optimal rank and number of clusters for running C-ZIPTF on real single-cell RNA sequencing dataset [54] of peripheral blood mononuclear cells from individuals with systemic lupus erythematosus and healthy groups

In this section, we explore the ability of C-ZIPTF to uncover intra-group sample heterogeneity in an unsupervised manner. To this end, we apply C-ZIPTF to a single-cell RNA sequencing dataset presented in a comprehensive study [54], which utilized multiplexed scRNA-seq (mux-seq) to profile over 1.2 million PBMCs from patients with systemic lupus erythematosus (SLE) and healthy controls. Importantly, amongst the SLE patients, several had an active lupus flare while others had a managed lupus. This is an important source of intra-group heterogeneity as we expect both shared and subgroup-specific gene expression programs to drive the transcriptional profiles of the two subgroups within the SLE patients. For our purposes, we downsampled the dataset to a subset of 8 SLE patients with flare, 8 SLE patients with managed disease, and 8 healthy controls. We also focused our analysis on essential immune cell types: CD4-positive alpha-beta T cells, CD8-positive alpha-beta T cells, classical monocytes, conventional dendritic cells, and NK cells, utilizing the cell type classifications provided in the original study.

**Fig. 8:**
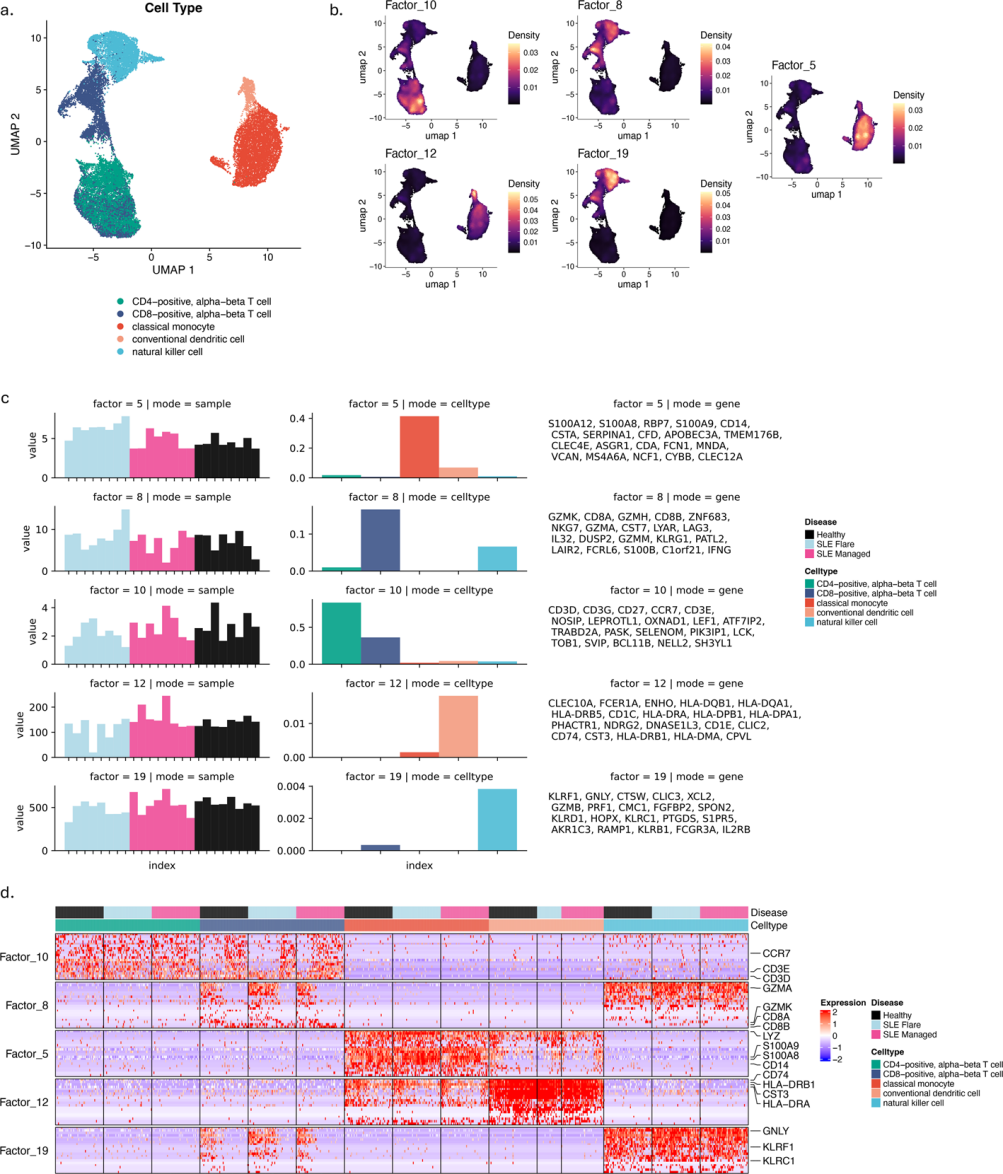
Cell type identity GEPs recovered by C-ZIPTF in the SLE dataset: **a** UMAP of immune cells in SLE dataset after downsampling, colored by cell type, **b** aggregated expression density of the top genes associated with each of the cell type identity factors, **c** 5 cell type identity factors recovered at rank 22 (each row represents a factor, and the first three columns display the three modes: sample, cell type, and gene, and the $y-$axis in the sample and cell type modes represent the loading of the sample or cell type on that factor. The gene mode exhibits the top 20 genes associated with the factor), **d** heatmap of normalized gene expression of the top genes associated with cell type identity factors in a randomly sampled set of single cells sorted by cell type and disease status

After downsampling, we retained 85,636 cells which we then used to create a pseudobulk tensor by summing raw counts for each sample, cell type, and gene. After removing genes that are not provided with HGNC symbols [49], as well as genes with a total count of less than 50, we ended up with a tensor of shape $24 \times 5 \times 13,525$ (samples $\times$ cell types $\times$ genes). We normalize the tensor such that each sample-cell type pair has a total of $10^6$ counts. We then performed tensor factorization with C-ZIPTF for ranks ranging from 2 to 30 with 30 random initializations each time. Figure 7 presents metrics employed in determining the optimal rank and number of clusters. We focused our downstream analysis on rank 22, which reaches the elbow point for explained variance (0.958) and peak for cophenetic correlation (0.953), Fig. 7a. We confirmed that with the rank set at 22, the gene latent factors obtained from multiple runs show a high silhouette score with 22 clusters, as depicted in Fig. 7b.

**Fig. 9:**
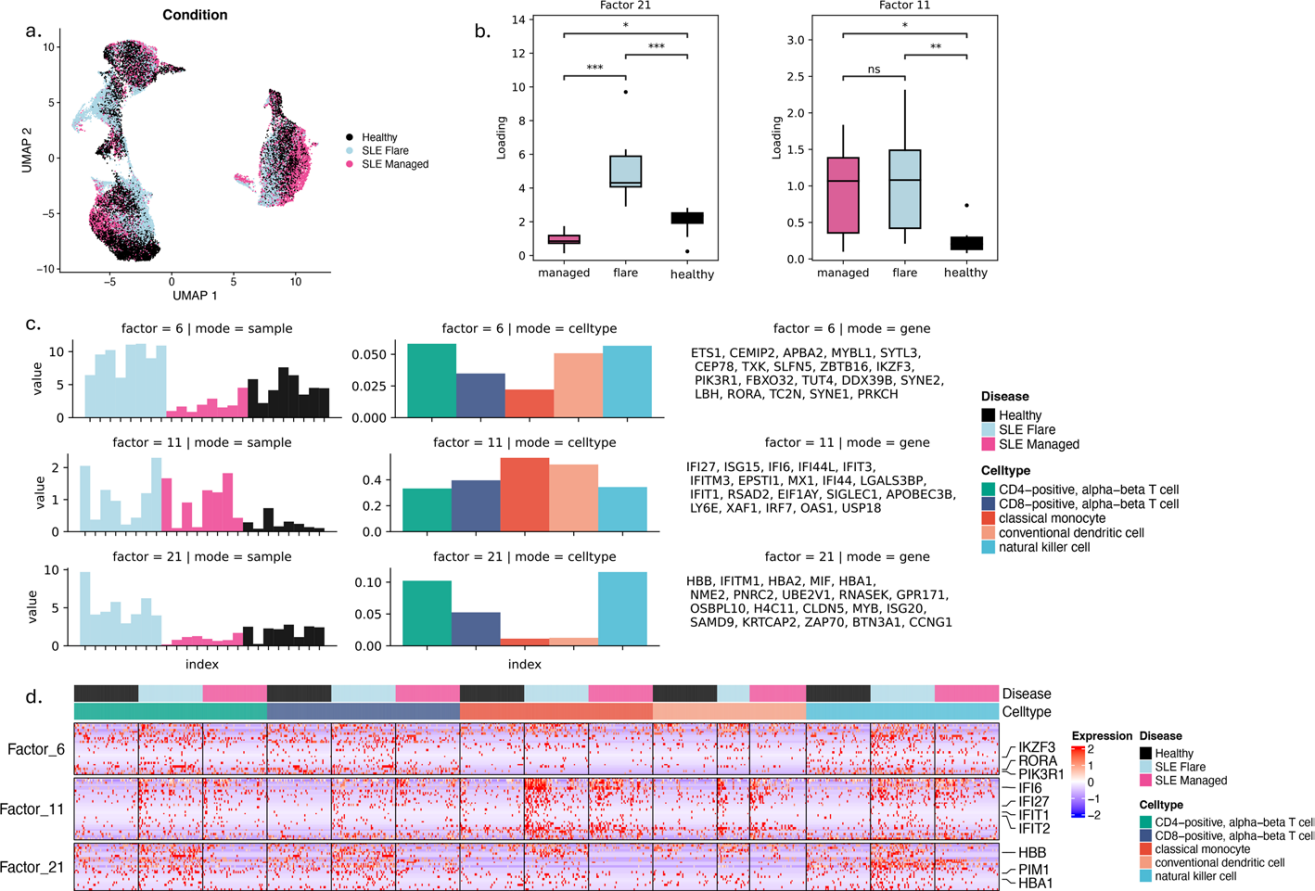
Condition specific GEPs recovered by C-ZIPTF in the SLE dataset: **a** UMAP of immune cells in SLE dataset after downsampling, colored by condition, **b** sample mode loading for factors 11 and 21 grouped by disease status, **c** 3 condition specific factors highlighted for rank 22 (Each row represents a factor, and the first three columns display the three modes: sample, cell type, and gene. The *y*-axis in the sample and cell type modes represent the loading of the sample or cell type on that factor. The gene mode exhibits the top 20 genes associated with the factor.), **d** heatmap of normalized gene expression of the top genes associated with the 3 condition specific factors in a randomly sampled set of single cells sorted by cell type and disease status

Once more, C-ZIPTF successfully identifies GEPs associated with cell type identities. Each of the 5 cell types included in the analysis had a corresponding factor capturing the GEP defining the cell type identity (Fig. 8b). The genes assigned to the factors after feature selection correspond to the established marker genes for each cell type [55]. To highlight a few, *CD14*, *LYZ* (classical monocytes), *CD74*, *HLA-DRB* (dendritic cells), *GNLY*, *KLRF1* (NK cells), *CD8A*, *GMZK* (CD8-positive T cells) are established marker genes of the corresponding cell types. Additionally, in cases where the identity GEPs are shared among different cell types, we observe the factor loading distributed across the corresponding cell types. For example, factor 10 which mainly represents CD4-positive T cell identity has genes representing pan T cell identity (*CD3D*, *CD3E*), therefore the loading for factor 10 is raised for CD8-positive T cells as well. Similarly, NK cells and CD8+ T cells share the cytotoxicity dimension of their identity GEPs (*GZMA*, *GZMK*) as reflected in the raised factor loading of the CD8-positive T cell identity program (factor 8) in NK cells (Fig. 8b-c). Moreover, as demonstrated in Fig. 8c, the genes associated with the identity factors are differentially expressed in the corresponding cell type cluster. The expression levels of the genes corresponding to factor 5 are high throughout the classical monocyte cluster and low elsewhere, and the same holds for the other identity factors. This is also reflected at the single-cell level as seen in Fig. 8d. The normalized expression of the top genes in each identity factor is predominantly expressed in the corresponding cell type and clearly has lower expression in single cells from other cell types.

**Fig. 10:**
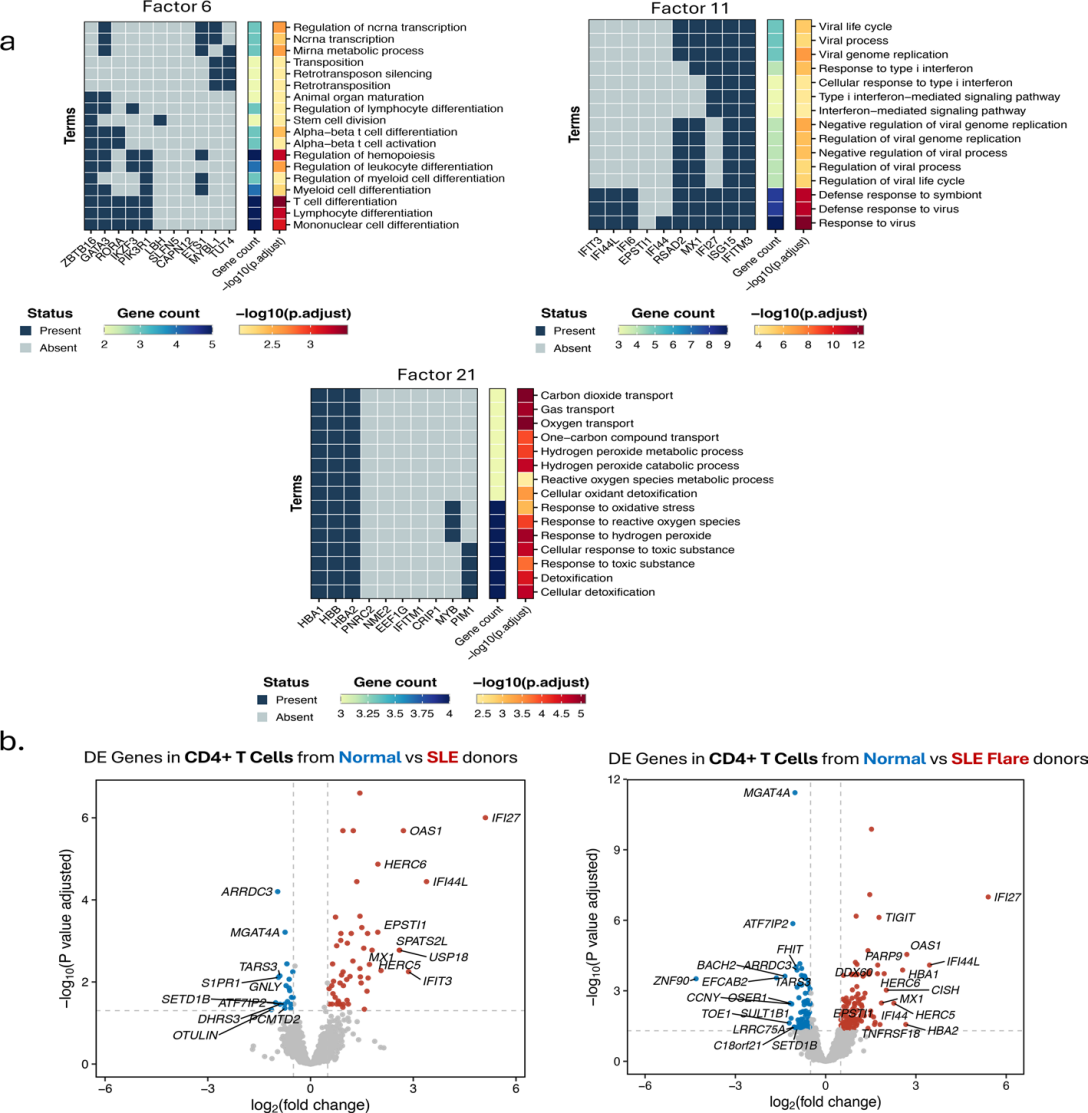
Condition specific GEPs recovered by C-ZIPTF compared to differential gene expression analysis: **a** top gene set enrichment analysis results for the 3 factors using Gene Ontology: Biological Process annotations, **b** results from a pseudobulk differential gene expression analysis are shown, with comparisons made between all SLE patients and healthy controls (on the left), and between patients with SLE flare and healthy controls (on the right)

Importantly, C-ZIPTF can also capture condition-specific GEPs and unravel nuanced expression patterns across immune cell types implicated in SLE pathogenesis in a manner that underscores the heterogeneity among SLE patients. In Fig. 9, we highlight the factors 6, 11, and 21, wherein the loadings in the sample dimension exhibit significant differences between SLE patients and healthy donors. Factor 11 captures a GEP that is overexpressed across both managed and flare SLE patients compared to healthy donors, Fig. 9b-c. Many of the genes corresponding to this factor such as IFI27, ISG15, IFITM3 are parts of the type 1 interferon signaling pathway normally observed in viral infections (Fig. 10a). Previous studies utilizing bulk RNA sequencing have noted a similar increase in interferon-related GEPs in patients with SLE [56]. On the other hand, factors 6 and 21 capture GEPs that are uniquely upregulated in SLE patients with an active lupus flare. The factor loadings on the sample dimension for these two factors are significantly higher in the donors with SLE flare than in healthy controls or donors with managed SLE. Both factors are predominantly represented in lymphoid cell types (T and NK cells), although factor 6 also shows a lower but notable loading for myeloid lineage cell types. Important genes associated with factor 21 include *HBA1, HBB, PIM1* which are implicated in biological processes such as Cellular Detoxification (Fig. 10a). Factor 6 on the other hand captures processes such as T Cell and Myeloid Cell Differentiation driven by genes including *RORA, IKZF3*, and *PIK3R1* (Fig. 10a).

The ability of C-ZIPTF to recover these subgroups of SLE patients without any a priori information underscores the advantages of the unsupervised factorization approach utilized in C-ZIPTF over standard practices such as DGE analysis. We explore this advantage by running differential expression analysis on the CD4-positive T cells using muscat [5]. We run DGE analysis first by grouping all SLE patients together and comparing them with healthy controls and then selecting only the SLE patients with an active flare and comparing them with the healthy controls. As demonstrated in Fig. 10b, cell type-specific differential expression analysis of all SLE donors versus healthy controls captures fewer differentially expressed genes compared to the SLE patients with an active flare versus healthy controls in CD4-positive T cells. The number of differentially expressed genes (DEGs) with an adjusted *p*-value less than 0.05 and a log fold change greater than 0.5 was 55 when comparing all SLE patients against healthy donors and 122 when comparing SLE patients with an active flare against healthy donors (Fig. 10b). This big difference underscores the obstacle sample heterogeneity poses to supervised methods of uncovering differential GEPs. Importantly, the condition specific factors recovered by C-ZIPTF expose both the shared (factor 11) and flare subgroup specific (factors 6 and 21) differential GEPs. Out of the top 20 genes associated with factor 11, 17 are part of the 55 significant DEGs that resulted from comparing CD4-positive T cells from all SLE patients with healthy donors. On the other hand, out of the top 20 genes associated with factor 6 and 21, only 1 gene was part of that set of 55 DEGs but 10 and 11 genes respectively were part of the set of 122 DEGs that resulted from comparing CD4-positive T cells from SLE flare patients with healthy donors. If information on disease activity (flare versus managed) were not available for this dataset, differential expression analysis would miss a lot of critical differentially expressed genes that are specifically upregulated in patients with SLE flare. Therefore, in scenarios where the source of intra-group heterogeneity is unknown, C-ZIPTF can highlight subgroups based on expression profiles and identify the GEPs driving heterogeneity that may be missed by supervised differential gene expression analysis.

### Computational efficiency

While Bayesian methods are known for their high computational demands, our strategy tackles these challenges by integrating Black Box Variational Inference [30] with tensor factorization. This fusion involves optimizing the variational objective stochastically, using Monte Carlo samples from the variational distribution to compute the noisy gradients efficiently. This approach significantly streamlines computations, enhancing the overall efficiency of Bayesian inference.

For runtime comparison, we assess three models: the MLE based NNCP-ALS, the Bayesian Poisson factorization model GPTF, and our proposed model ZIPTF. Further implementation details are provided in Section Implementation of baseline methods. We employ the Black-Box Variational Inference algorithm for both Bayesian approaches, namely GPTF and ZIPTF. We generate tensors using known factors and Poisson noise with zero inflation. To generate $\mathcal {T'}$ of given size $I\times J \times K$, a sequence of steps is followed. Initially ${\mathcal {T}}$ is formulated as ${\mathcal {T}}= \sum _{i=1}^{R} a_{i} \otimes b_i \otimes c_i$ such that $A=[a_1a_2 \ldots a_R] \in {\mathbb {R}}_{+}^{I\times R},~B=[b_1b_2\ldots b_R] \in {\mathbb {R}}_{+}^{J\times R}$, and $C=[c_1 c_2 \ldots c_R] \in {\mathbb {R}}_{+}^{K\times R}$ with elements drawn from a Gamma distribution with shape $\alpha =1$ and rate $\beta =0.3$, and rank $R=10.$ Following this, ${\mathcal {T}}'$ is generated by sampling from a ZIP distribution with mean ${\mathcal {T}}$ and a given probability of extra zeros $\Phi =0.5.$

We created 100 tensors of given sizes using the aforementioned steps. Each factorization algorithm was executed 20 times, and the running times were recorded. The average and standard deviation of the recorded running times were computed across all 20 executions for each algorithm applied to the 100 tensors of specified sizes. The findings are detailed in Table 1.

**Table 1:** Average running time ± standard deviation (in seconds) from 20 runs of each algorithm on 100 tensors of given sizes

Tensor size	NNCP-ALS	GPTF	ZIPTF
$10 \times 20 \times 30$	$0.595s \pm 0.008s$	$1.039s \pm 0.005s$	$1.685s \pm 0.014s$
$10 \times 20 \times 300$	$0.725s \pm 0.023s$	$1.551s \pm 0.011s$	$2.773s \pm 0.016s$
$10 \times 20 \times 3000$	$1.542s \pm 0.076s$	$3.426s \pm 0.019s$	$5.239s \pm 0.084s$
$10 \times 20 \times 30000$	$9.109s \pm 0.651s$	$19.640s \pm 0.255s$	$37.687s \pm 0.599s$

## Discussion

Zero-inflated count data is a common phenomenon in a wide range of fields, including genomics, finance, risk management, healthcare, and social sciences. However, traditional tensor factorization methods have limited effectiveness when dealing with zero-inflated data, often yielding inaccurate and unstable results across runs with different initializations. To overcome these challenges, we propose ZIPTF, a Bayesian tensor factorization model that is specifically tailored to zero-inflated count data. Additionally, we introduce a generic meta-analysis framework for consensus-driven tensor factorization. By combining these two approaches, we develop a novel method called C-ZIPTF that achieves both high accuracy and stability, and outperforms state-of-the-art baselines on synthetic and real data. Our proposed method provides a useful tool for researchers in various fields to gain deeper insights into their data.

Another crucial aspect to highlight is the remarkable degree of versatility of the model. By combining the Black Box variational inference with tensor factorization, we’ve introduced a methodology that can be readily tailored to the specific data distribution within a tensor. While we initially applied zero-inflated Poisson for factorizing scRNA-seq data, which is a suitable starting point considering the dropout challenges in such data [4], straightforward extensions of this approach would enable us to create more refined models, including the summation of Poisson distributions and various other mixture models.

This paper mainly focuses the model’s applications in the context of multi-sample/multi-condition single-cell data. However, owing to the inherent multidimensional characteristics of multi-omics datasets, proposed models also well-suited for seamlessly integrating and collectively analyzing multi-omics data derived from diverse sources.

Another promising area where ZIPTF could be employed is the mutational signature analysis. This analysis enhances our understanding of tumorigenesis by identifying key mutational processes, which informs cancer classification, prognosis, and personalized treatment decisions based on genetic profiles [57, 58]. However, analyzing mutation data presents challenges, including sparsity among other factors. Addressing these challenges, zero-inflated models are commonly used [59]. For future research, applying techniques like ZIPTF to mutation data holds promise for improving mutational signature acquisition and analysis.

## Limitations

The Bayesian approach for tensor factorizations offers several other advantages over maximum likelihood estimation-based methods for tensor factorization. These include the ability to incorporate prior knowledge, perform model selection, and quantify uncertainty in the parameter estimates. However, it is important to note that Bayesian methods can be computationally expensive and require careful specification of prior distributions, which may require expert knowledge. Moreover, the tensor methods discussed in this paper rely on a multilinear factorization form and may be inadequate for capturing more complex, nonlinear relations in the data. To overcome this limitation, one possible solution is to integrate a kernelized approach into the factorization. In future work, we plan to focus on the careful design of kernel functions that would enable us to effectively capture nonlinear patterns in the data.

In the context of applying the model to scRNA-seq data, ZIP models handle zero counts, but they do not inherently address the systematic patterns of the missing data that could be present in scRNA-seq due to factors beyond dropout events. When addressing the missing-not-at-random (MNAR) phenomenon in scRNA-seq data, it becomes essential to incorporate specific biological or technical covariates that might influence the missingness patterns [60]. In this context, enhancing Bayesian frameworks to integrate prior knowledge concerning the missing data mechanism can offer a more robust approach to model and imput MNAR patterns, resulting in enhanced analysis outcomes.

## Implementation of ZIPTF and C-ZIPTF

We present a Python implementation of a versatile Bayesian Tensor Factorization method using Variational Inference. Our implementation leverages Pyro [36], a probabilistic programming framework built on PyTorch. The BayesianCP class inherits from torch.nn.Module and offers functionalities for model fitting and summarizing the posterior distribution of factor matrices. During model fitting, Stochastic Variational Inference (SVI) is employed with an Adam optimizer [29, 61]. The current implementation supports three models: Zero Inflated Poisson model (ZIPTF), a Gamma Poisson model (GPTF) [13], and a Truncated Gaussian model (TGTF) [12].

### Implementation of baseline methods

As mentioned in Section Implementation of ZIPTF and C-ZIPTF, we utilize the same implementation for other Bayesian tensor factorization approaches (Gamma Poisson Bayesian Tensor Factorization and Truncated Gaussian Bayesian Tensor Factorization) as we do for the ZIPTF method. You can find the code at the following URLs: https://github.com/klarman-cell-observatory/scBTF and https://github.com/klarman-cell-observatory/scbtf_experiments. For the remaining baseline methods used in our comparisons we use the following implementations:

- *Non-negative Matrix Factorization (NMF)*: We use the Python implementation available in the scikit-learn package, which can be found at the following URL: https://scikit-learn.org/stable/modules/generated/sklearn.decomposition.non_negative_factorization.html.
- *Consensus Non-negative Matrix Factorization (cNMF)*: We employ the Python implementation as detailed in [20], which is accessible on GitHub at the following URL: https://github.com/dylkot/cNMF/tree/master.
- *Non-negative CP via Alternating-Least Squares (NNCP-ALS)*: We utilize the Python implementation available within the Tensorly package, which can be accessed at this URL: http://tensorly.org/stable/modules/generated/tensorly.decomposition.non_negative_parafac_hals.html

### Simulation details

We use a Python adaptation of the Splatter [45] statistical framework given in [20] to simulate single-cell RNA-Seq data. The core of the simulation is a Gamma-Poisson distribution used to generate a cell-by-gene count matrix. While the original Splatter framework supports the simulation of both expression outlier genes and technical dropout (random knockout of counts), the Python adaptation in [20] only keeps outlier expression simulation. Since our method is specifically adapted to handle dropout noise in single-cell data, we add back the modeling of dropout to the Python adaptation. Specifically, after sampling counts from a Poisson distribution, we simulate dropout noise by calculating the probability of a zero for each gene from its mean expression and using that to randomly replace some of the simulated counts with zeros employing a Bernoulli distribution as described in [45].

The distribution of expression values prior to incorporating differential expression was determined based on parameters estimated from a random sample of 8000 cells from an organoid dataset as described in [20]. Specifically, the library size of a cell is sampled from a Lognormal distribution derived from a Normal distribution with a mean of 7.64 and a standard deviation of 0.78. The mean expression of a gene is sampled from a Gamma distribution with a mean of 7.68 and a shape of 0.34. With the probability of 0.00286, a gene will be an outlier from this Gamma distribution and will instead be sampled from a Lognormal distribution derived from a Normal distribution with a mean of 6.15 and standard deviation of 0.49. Additionally, we set a 5% doublet rate. Doublets are formed by randomly sampling a pair of cells, combining their gene counts, and downsampling such that the total count equals the larger of the two.

## Data Availability

All data generated or analyzed in this study, along with the code, is available at the following links https://github.com/klarman-cell-observatory/scBTF and https://github.com/klarman-cell-observatory/scbtf_experiments.
